# Adipose Tissue Sex Steroids in Postmenopausal Women With and Without Menopausal Hormone Therapy

**DOI:** 10.1210/clinem/dgae458

**Published:** 2024-07-10

**Authors:** Natalia Hetemäki, Alexandra Robciuc, Veera Vihma, Mikko Haanpää, Esa Hämäläinen, Matti J Tikkanen, Tomi S Mikkola, Hanna Savolainen-Peltonen

**Affiliations:** Obstetrics and Gynecology, University of Helsinki and Helsinki University Hospital, FIN-00029 HUS Helsinki, Finland; Obstetrics and Gynecology, University of Helsinki and Helsinki University Hospital, FIN-00029 HUS Helsinki, Finland; Department of General Practice and Primary Health Care, University of Helsinki, FIN-00014 Helsinki, Finland; HUSLAB, Helsinki University Hospital, FIN-00029 HUS Helsinki, Finland; Department of Clinical Chemistry, University of Helsinki, FIN-00029 HUS Helsinki, Finland; Department of Clinical Chemistry, University of Eastern Finland, FIN-70210, Kuopio, Finland; Heart and Lung Center, University of Helsinki and Helsinki University Hospital, FIN-00029 HUS Helsinki, Finland; Obstetrics and Gynecology, University of Helsinki and Helsinki University Hospital, FIN-00029 HUS Helsinki, Finland; Obstetrics and Gynecology, University of Helsinki and Helsinki University Hospital, FIN-00029 HUS Helsinki, Finland

**Keywords:** adipose tissue, sex steroids, postmenopausal, menopausal hormone therapy

## Abstract

**Context:**

The decrease in serum estrogens after menopause is associated with a shift from a gynoid to an android adipose tissue (AT) distribution. Menopausal hormone therapy (HT) mitigates this change and accompanying metabolic dysfunction, but its effects on AT sex steroid metabolism have not been characterized.

**Objective:**

We studied effects of HT on subcutaneous and visceral AT estrogen and androgen concentrations and metabolism in postmenopausal women.

**Design, setting, patients, and interventions:**

Serum and subcutaneous and visceral AT from 63 postmenopausal women with (n = 50) and without (n = 13) per oral HT were analyzed for estrone, estradiol, progesterone, testosterone, androstenedione, dehydroepiandrosterone, and serum estrone sulfate using liquid chromatography-tandem mass spectrometry. Steroid sulfatase activity was measured using radiolabeled precursors. mRNA expression of genes encoding sex steroid-metabolizing enzymes and receptors was performed using real-time reverse transcription quantitative polymerase chain reaction.

**Results:**

HT users had 4- to 7-fold higher concentrations of estrone and estradiol in subcutaneous and visceral AT, and 30% lower testosterone in visceral AT compared to nonusers. Estrogen-to-androgen ratios were 4- to 12-fold higher in AT of users compared to nonusers of HT. In visceral AT, estrogen-to-androgen ratios increased with HT estradiol dose. AT to serum ratios of estrone and estradiol remained high in HT users.

**Conclusion:**

Higher local estrogen to androgen ratios and high AT to serum ratios of estrogen concentrations in HT users suggest that HT may significantly influence intracrine sex steroid metabolism in AT; these local changes could be involved in the preventive effect of HT on menopause-associated abdominal adiposity.

Extragonadal production of sex steroids is highlighted after the cessation of ovarian estrogen synthesis at menopause. Most circulating estrogens in postmenopausal women are produced in peripheral tissues, particularly in adipose tissue (AT) (1-3), from androgen precursors predominantly produced in the adrenal glands and ovaries (4). Androgen and estrogen concentrations in AT exceed circulating levels (3, 5-7), reflecting the role of AT as an important reservoir and site of metabolism of sex steroids.

Sex steroids also regulate body AT deposition and function (8). At menopause, circulating levels of androgens decrease less than those of estrogens because androgens continue to be produced by the postmenopausal ovaries and through the conversion of adrenal precursor steroids, such as dehydroepiandrosterone (DHEA), dehydroepiandrosterone sulfate (DHEAS), and androstenedione (A_4_), to active androgens in peripheral tissues (8-10). A lower estrogen to androgen ratio in the circulation has been associated with an increased relative amount of visceral compared to subcutaneous AT (11). Moreover, adiposity may correlate positively with testosterone (T) levels in serum from postmenopausal women (12). Visceral, or android, obesity is associated with an unfavorable inflammatory marker and adipokine profile (13), leading to AT dysfunction and metabolic disturbances such as insulin resistance, cardiovascular disease, metabolic syndrome, and an increased risk for postmenopausal hormone receptor-positive breast cancer (14-18).

Menopausal hormone therapy (HT) is indicated for the treatment of bothersome menopausal symptoms (19). In addition to symptom relief, HT mitigates the increase in android or visceral adiposity during the menopausal transition (20-23). Among favorable metabolic effects of HT are a reduced risk for type 2 diabetes (24) and a healthier adipokine/cytokine profile compared to nonusers of HT (25). HT initiated shortly after menopause has been associated with cardioprotective effects (26, 27). To our knowledge, no studies to date have addressed the question of how postmenopausal HT affects the concentrations and metabolism of sex steroids in AT.

To explore this question, we measured local subcutaneous and visceral AT and circulating estrogen, androgen, and progesterone concentrations by specific liquid chromatography-tandem mass spectrometry (LC-MS/MS) in postmenopausal women with and without per oral HT. We also studied the production of estrone (E_1_) from estrone sulfate (E_1_S) by the steroid sulfatase (STS) enzyme. Relative gene mRNA expression levels of key estrogen- and androgen-metabolizing enzymes (STS; aromatase; 17*β*-hydroxysteroid dehydrogenase types 1, 2, 7, and 12), as well as mRNA expression levels of genes encoding estrogen receptors α and β (*ESR1*, *ESR2*), the androgen receptor (*AR*) and progesterone receptors (*PRb, PRa/b*) were measured in AT using real-time reverse transcription quantitative PCR.

## Materials and Methods

### Subjects and Study Design

Samples of abdominal subcutaneous and visceral AT were obtained during abdominal or laparoscopic surgery from postmenopausal women (n = 63) at the Department of Obstetrics and Gynecology, Helsinki University Hospital. Study subjects gave their signed informed consent before surgery to participate in the study, and the study protocol was accepted by the Ethics committee of the Helsinki University Hospital.

Indications for surgery were nonmalignant and included: uterine fibroids (n = 22), benign endometrial hyperplasia (n = 2), ovarian cysts (n = 25), chronic salpingitis and oophoritis (n = 3), partial or total vaginal prolapse (n = 10), and dyspareunia (n = 1). Before surgery, blood samples were drawn, and serum was separated by centrifugation within 1 hour. The serum samples were stored in the deep freezer (at −80 °C) until analysis. AT samples ranging from 200 mg to 5 g each, were snap-frozen in liquid nitrogen, and stored at −80 °C until analysis.

Fifty women used per oral HT (HT+) and 13 women were nonusers of HT (HT−). Postmenopausal status was defined as at least 1 year since date of last menses and confirmed by measuring the level of FSH (≥30 IU/L for nonusers of HT, Helsinki University Hospital laboratory). One woman had an FSH level of 25.1 IU/L. Her age was 80 years at the time of surgery, and she reported a time of 36 years since the date of her last menses and was therefore included in the study. Anthropometric data on body mass index (BMI), waist circumference, hip circumference, waist/hip ratio, height, and body adiposity index (BAI) were recorded and calculated and used as estimates of adiposity. BAI was calculated using the formula: BAI = hip circumference (cm)/[height (m) × square root (height)]—18 (28). Two women in the HT+ group and 3 in the HT− group had metabolic syndrome, defined as waist circumference >90 cm and medication for 2 or 3 conditions out of hypertension, hypercholesterolemia, and diabetes.

According to current best practice, only hysterectomized women can take estrogen-only therapy (ET), whereas women with an intact uterus are prescribed estrogen-progestin therapy (EPT) (19). The HT+ group (n = 50) consisted of 17 ET users (estradiol [E_2_] valerate or E_2_ hemihydrate), and 33 EPT users (E_2_ + 52 mg levonorgestrel-releasing intrauterine system (n = 5); E_2_+ norethisterone acetate (n = 7); E_2_+ medroxyprogesterone acetate (n = 11); or E_2_+ dydrogesterone (n = 10). In the HT+ group, the daily dose of E_2_ ranged from 0.5 to 2 mg (0.5 mg, n = 4; 1 mg, n = 39; 2 mg, n = 6) and further analyses were also conducted according to the E_2_ dose, except for 1 study participant whose dose was not traceable from the patient charts. In all, there were no differences in the sex steroid concentration or gene mRNA expression levels between users of different progestins, or between users of ET and EPT (data not shown). Between-group comparisons are therefore presented between HT+ and HT−.

Women with and without HT were comparable in their primary clinical characteristics, except for the concentration of SHBG, which was higher in users of HT, and BMI, which was slightly lower in users of HT (Table 1). In all study subjects, BAI correlated strongly with other anthropometric parameters: BMI (*r* = 0.78, *P* < .001, n = 58), waist circumference (*r* = 0.90, *P* < .001, n = 58), and waist/hip ratio (*r* = .79, *P* < .001, n = 58).

**Table 1.** Clinical characteristics of postmenopausal women without and with HT

Clinical characteristics	HT−n = 13	HT+ n = 50	*P*-value*^a^*
Age, y	62 (55-80)	62 (51-81)	.87
Years since menopause	10 (3-36)	11 (1-29)	.75
Body mass index, kg/m^2^	28 (22-36)	26 (20-34)	.**03**
Waist/hip ratio	0.89 (0.79-1.02)	0.87 (0.74-1.05)	.33
Waist circumference, cm	97 (81-120)	90 (70-115)	.10
Body adiposity index	30 (21-40)	25 (15-37)	.06
Serum FSH, IU/L	49.8 (25.1-71.4)	38.0 (1.4-110.4)	.09
Serum SHBG, nmol/L	51 (16-89)	72 (28-176)	**<**.**001**

Data are expressed as median (range). Boldface indicates statistical significance.

Abbreviations: HT, hormone therapy; HT−, non-users of HT; HT+, users of HT.

^*a*^Independent-samples Mann-Whitney *U* test.

### Quantitation of E_1_, E_1_S, and E_2_ in Serum

Serum E_1_ and E_2_ were quantitated by LC-MS/MS as previously described (29, 30), with some modifications. Briefly, assay calibrators of 0.0 to 1000 pmol/L E_1_ (Vetranal, Sigma–Aldrich, St Louis, MO) and 0.0 to 1275 pmol/L E_2_ (Sigma–Aldrich) were prepared in water:methanol (1:1, volume-to-volume ratio [v/v]). To 0.3 mL of calibrator or serum samples, 30 µL of internal standard was added containing 3  nmol/L ^13^C_3_-E_1_ and 3  nmol/L ^13^C_3_-E_2_ (IsoSciences, Ambler, PA) in water:methanol (19:1, v/v). A serum sample or assay calibrator with internal standard was extracted with 1 mL of diethyl ether (DEE). The DEE phase was transferred into a vial and evaporated to dryness after centrifugation. A total of 0.3 mL of 0.1% ammonia water (Sigma-Aldrich) and 1 mL of DEE were added to the residue followed by a second extraction and evaporation of the DEE phase after centrifugation. The serum sample or assay calibrator residue was dissolved in 0.125 mL of water:methanol (1:1, v/v). A total of 0.1 mL of calibrators and sample extracts were analyzed by LC-MS/MS using an 8-point calibration curve. The mobile phase was a linear gradient consisting of 40 µmol/L ammonium fluoride in water (A) and methanol (B) at a flow rate of 0.3 mL/min. The limit of quantification (LOQ) for E_1_ and E_2_ was 5 pmol/L with signal to noise ratios of 10 or higher. For purposes of statistical analysis, a value of 1 pmol/L was assigned to samples whose concentration was <5 pmol/L.

Serum E_1_S was quantitated using LC-MS/MS as described (6). The LOQ for E_1_S in serum was 0.1 nmol/L.

### Quantitation of A_4_, T, DHEA, and Progesterone in Serum

Serum A_4_, T, DHEA, and progesterone levels were quantitated by LC-MS/MS as previously described (30), with the modification that the serum sample and calibrator volumes were 0.3 mL and the linear gradient for DHEA was 0 minutes 50% B, 10 to 14 minutes 100% B, and 14.5 to 22 minutes 50% B. LOQ were 10 pmol/L for A_4_, T, and progesterone, and 100 pmol/L for DHEA.

### Quantitation of E_1_ and E_2_ in AT

Subcutaneous and visceral AT E_1_ were quantitated by LC-MS/MS as described (31), with some modifications. Briefly, assay calibrators of 0.0 to 1000 pmol/L E_1_ were prepared in water:methanol (1:1, v/v). After sample homogenization, 30 µL of internal standard containing 10 nmol/L ^13^C_3_-E_1_ (IsoSciences) in water:methanol (1:1, v/v) was added to samples and controls, which were then extracted and purified as described (31). An aliquot 0.25 mL was evaporated to dryness and sent for further processing and analysis of E_1_ with LC/MS-MS. E_1_ and corresponding ^13^C_3_-E_1_-labelled internal standard were detected for duplicate quantitation as described previously for serum E_1_. Interassay imprecision was 2% and 3% for pooled male serum used as control (Sigma-Aldrich; with 180 and 540 pmol/L of added exogenous E_1_) in 12 consecutive assays. The LOQ for E_1_ in AT was 10 pmol/L.

E_2_ concentration in subcutaneous and visceral AT was determined using LC-MS/MS as described (6). Interassay imprecision was 5% for pooled male serum (Sigma-Aldrich) and 2% and 5% for pooled male serum used as control (Steraloids Inc, Newport, RI; with 112 and 260 pmol/L of added exogenous E_2_), in 14 consecutive assays, and 19% and 6% for 2 pools of female AT in 7 consecutive assays each. The LOQ for E_2_ in AT was 10 pmol/L.

### Quantitation of A_4_, T, DHEA, and Progesterone in AT

Extraction and purification of DHEA, A_4_, T, and progesterone from subcutaneous and visceral AT was carried out as previously described for DHEA (32), with the following modifications: 250 mg of AT was homogenized in distilled water and 30 µL of internal standard containing 0.1 µmol/L ^13^C_3_-A_4_, 0.2 µmol/L ^13^C_3_-T, 0.2 µmol/L ^13^C_3_-DHEA, and 0.4 µmol/L ^13^C_3_-P_4_ (IsoSciences) in water:methanol (1:1, v/v) were added. The samples were extracted 4 times with 4 mL of diethyl ether. After evaporation, the samples were dissolved in 0.3 mL of hexane and applied to Sephadex LH-20 columns (0.5 × 5 cm in hexane) in 3 0.3-mL aliquots of hexane. The column was washed twice with 5 mL hexane, and DHEA, A_4_, T, and progesterone were eluted with 6 mL of hexane:chloroform (1:1, v:v). Organic solvents were evaporated to dryness under nitrogen flow, and the steroid fractions were analyzed by LC-MS/MS as described for serum (30). The LOQ were 10 pmol/L for A_4_, T, and progesterone, and 100 pmol/L for DHEA.

Intra-assay variation was 3% for T, 2% for A_4_, 7% for DHEA, and 3% for progesterone, in 8 parallel determinations of pooled female AT. Interassay imprecision in pooled female and male serum used as control was 3% and 4% for T, 2% and 3% for A_4_, 6% and 4% for DHEA, and 4% and 3% for progesterone in 7 consecutive assays. Interassay imprecision for pooled female AT used as controls was 16% for T, 5% for A_4_, 6% for DHEA, and 8% for progesterone in 7 consecutive assays.

To determine analytical recovery, T, A_4_, DHEA (Sigma–Aldrich), and progesterone (IsoSciences) were added to 200 mg of pooled female AT at low, medium, and high concentrations. Pooled AT samples with no added analytes served as controls. A total of 3 nmol/L ^13^C_3_-T, 3 nmol/L ^13^C_3_-A_4_, 12 nmol/L ^13^C_3_-progesterone, and 12 nmol/L ^13^C_3_-DHEA (IsoSciences) were added as internal standards to samples and controls. The results of triplicate determinations are shown in Table 2.

**Table 2.** Analytical recovery of T, A_4_, DHEA, and progesterone added to pooled AT

	T	A_4_	DHEA	Progesterone
Low	99% (97%-102%)	107% (105%-110%)	98% (92%-104%)	96% (80%-105%)
Medium	103% (101%-106%)	99% (99%-100%)	94% (91%-96%)	96% (93%-99%)
High	99% (99%-100%)	97% (92%-99%)	97% (90%-100%)	98% (91%-102%)

The results are calculated (observed/added)×100 (range). Low, 0.1 nmol/L T, 1 nmol/L A_4_, 5 nmol/L DHEA, and 2 nmol/L progesterone; medium, 0.5 nmol/L T, 5 nmol/L A_4_, 15 nmol/L DHEA, and 8 nmol/L progesterone; high, 5 nmol/L T, 20 nmol/L A_4_, 50 nmol/L DHEA, and 40 nmol/L progesterone.

Abbreviations: A_4_, androstenedione; DHEA, dehydroepiandrosterone; T, testosterone.

For evaluation of possible matrix effects (ion suppression), 6 200-mg samples of pooled female AT were prepared for LC-MS/MS analysis as described previously. A total of 0.5 nmol/L T, 5 nmol/L A_4_, 15 nmol/L DHEA (Sigma–Aldrich), and 8 nmol/L progesterone (IsoSciences) were added to 3 samples, and 3 nmol/L ^13^C_3_-T, 3 nmol/L ^13^C_3_-A_4_, 12 nmol/L ^13^C_3_-progesterone, and 12 nmol/L ^13^C_3_-DHEA (IsoSciences) to the remaining 3 samples. The same concentrations of analytes and internal standards were added to control samples of water:methanol (1:1, v/v). The recoveries of added analytes and internal standards were calculated as percentages of control samples analyte peak areas. The recovery percentages of added analytes were 80% (72%-86%) for T, 76% (64%-83%) for A_4_, 49% (45%-53%) for DHEA, and 77% (66%-89%) for progesterone; for internal standards, the corresponding recovery percentages were 82% (80%-83%) for T, 78% (76%-79%) for A_4_, 55% (44%-83%) for DHEA, and 89% (85%-92%) for progesterone.

### Estrogen to Androgen Ratios

The ratio of concentrations of E_2_ to T, E_1_ to A_4_, and E_2_ + E_1_ to T + A_4_ in AT were calculated to evaluate the estrogen to androgen balance within AT. These ratios can also be interpreted as indirect estimates of aromatase activity (33).

### Quantitation of Serum Leptin, Adiponectin, and Interleukin-6

Leptin, adiponectin, and IL-6 levels in serum were assayed using commercial ELISAs: Human Leptin ELISA Kit—Quantikine (Catalog # DLP00, RRID:AB_2783014; R&D Systems Inc, Minneapolis, MN), Human IL-6 DuoSet ELISA Kit (Catalog # DY206, RRID:AB_2814717; R&D Systems), and Adiponectin Human ELISA (Competitive) (Catalog # RD195023100, RRID:AB_2909450; BioVendor Group, Czech Republic) as instructed by the manufacturers.

### Activity Assay for STS in Converting E_1_S to E_1_

AT samples of ∼200 mg were homogenized in 1 mL of 0.1 mol/L Tris-HCl (pH, 7.5), and labelled with 5.3 × 10^5^ dpm (or 6.0 pmol per 200 mg AT; weighted mean) for the HT+ group; and with 5.6 × 10^5^ dpm (or 6.3 pmol per 200 mg AT) for the HT− group, of purified tritium-labeled E_1_S ([6,7-^3^H(N)], specific activity 1.48 TBq/mmol, Perkin Elmer). Samples and controls ([^3^H]-E_1_S in Tris-HCl) were incubated for 3 hours at +37 °C, the incubation mixture was extracted and purified, and [^3^H]-E_1_ was determined as described previously (6). STS activity was determined as [^3^H]-E_1_ (nmol) formed from [^3^H]-E_1_S per AT mass (kg) per time of incubation (h).

### RNA and cDNA Preparation, and mRNA Quantitation

Relative mRNA expression levels of *STS, CYP19A1, HSD17B1*, *HSD17B2*, *HSD17B7*, and *HSD17B12*, *ESR1, ESR2, AR, PRb*, and *PRa/b* were measured using quantitative real-time PCR. The mRNA was extracted and purified from AT as described elsewhere (34).

The quantity and quality of total mRNA was analyzed by Nanodrop spectrophotometer (Thermo-Fisher Scientific, Waltham, MA). The RNA was reverse transcribed into cDNA using SuperScript VILO kit (Thermo-Fisher Scientific). A total 5 ng of cDNA was used for the specific amplification of *STS, CYP19A1, ESR1, ESR2, HSD17B1*, *HSD17B7*, *HSD17B12*, *AR, PRb*, and *Pra/b*, and 10 ng for *HSD17B2*. The geometric mean of 3 reference genes, *importin 8 (IPO8), GAPDH*, and *14-3-3 protein zeta/delta (YWHAZ)*, was used for normalization. The data are presented as fold change compared to control. We used commercial primers for *ER1*, *ER2*, *HSD17B1*, and *HSD17B2* (Bio-Rad, Hercules, CA); the primer sequences for the other gene targets are included in Supplementary Table 1 (35).

### Other Analyses

Serum levels of FSH and SHBG were measured as described (36, 37) using accredited methods (Helsinki University Hospital laboratory).

Free T concentration in serum was calculated using Anderson's formula (38, 39):

Free T [pmol/L] = total T [nmol/L] × [2.28-1.38 × log(SHBG [nmol/L]/10)] × 10.

Free E_2_ was calculated using the formula: Free E_2_ [nmol/L] = [10^(−0.003×SHBG [nmol/L]+0.389)^/100] × E_2_ [nmol/L] (40).

### Statistical Analysis

Statistical analysis was carried out using IBM SPSS Statistics 28.0 software. The normality of distributions was evaluated with the Shapiro-Wilk test. For nonparametric variables, the Wilcoxon signed-rank test was used for pairwise comparisons, and the independent-samples Mann-Whitney *U* test and the independent-samples Kruskal-Wallis tests for between-group analyses. For parametric variables, between-group differences were assessed using Student *t* test.

To evaluate the possible effect of the difference in BMI between nonusers and users of HT, each HT− group subject (n = 13) was matched with 2 HT+ group subjects with similar BMI, and between-group analyses were repeated for comparison.

Correlations were analyzed using Spearman's nonparametric correlation. Data are presented as median (interquartile range) or median (range), unless otherwise stated. The level of statistical significance was *P* < .05.

## Results

### Sex Steroid Concentrations in AT and Serum

**Figure 1.**
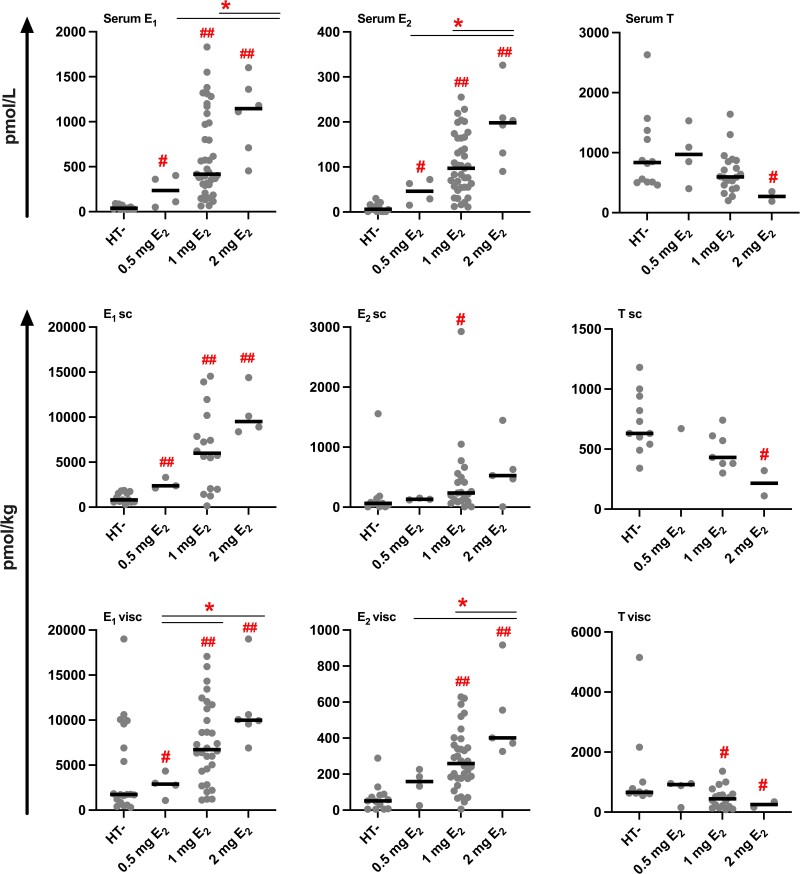
Sex steroid concentrations in serum and AT according to dose of E_2_ in users of HT. The black line represents the median. #*P* < .05; ##*P* < .001, compared to HT−; *P < .05, compared to 1 mg or 2 mg E_2_ (independent-samples Mann-Whitney *U* test).

Abbreviations: E_2_, estradiol; HT−, nonusers of HT; E_1_, estrone; Sc, subcutaneous adipose tissue; T, testosterone; Visc, visceral adipose tissue.

AT and serum concentrations of estrogens were both significantly higher in the HT+ compared to the HT− group (Table 3, Fig. 1). AT estrogen levels were 4 to 7 times higher in users of HT compared to nonusers. In women using HT, the concentration of E_1_ was higher in visceral compared to subcutaneous AT (*P* < .01, n = 23; Wilcoxon signed-rank test). The serum concentration of E_1_S was 12 times greater, E_1_ was 11 times greater, and E_2_ was 16 times higher in the HT+ compared to the HT− group. The AT to serum concentration ratios of E_1_ and E_2_ were high in both groups, in HT users especially for E_1_ (14 and 16 in subcutaneous and visceral AT, respectively).

**Table 3.** Concentrations of serum and AT estrogens and androgens in users and non-users of HT

		HT−n = 13	HT+ n = 50	*P*-value*^a^*
Serum, pmol/L				
E_1_S		1200 (600-1750)	14 600 (4625-27 300)*^b^*	**<.001**
E_1_		39 (27-70)	427 (306-1095)	**<.001**
E_2_		6 (1-18)	94 (55-168)	**<.001**
Free E_2_		0.11 (0.02-0.30)	1.26 (0.75-2.26)*^c^*	**<.001**
Free E_2_, %		1.7 (1.6-1.9)	1.5 (1.2-1.6)*^c^*	**<.001**
T		835 (510-1333)*^d^*	600 (400-880)*^e^*	.17
Free T		10.8 (7.7-17.3)*^d^*	6.1 (4.6-9.6)*^f^*	**<.01**
Free T, %		1.3 (1.2-1.5)*^d^*	1.1 (0.9-1.2)*^f^*	**<.001**
A_4_		2500 (1500-3100)*^g^*	2300 (1500-3100)*^e^*	.96
DHEA		7500 (4800-12 300)*^d^*	7200 (5100-11 800)*^e^*	.96
Progesterone		120 (100-210)*^d^*	130 (80-160)*^e^*	.79
Adipose tissue, pmol/kg				
E_1_	Sc	814 (586-1636)	5989 (2192-9807)*^h^*	**<**.**001**
	Visc	1629 (525-1748)	6731 (2950-10 192)*^i^*	**<**.**001**
E_2_	Sc	63 (5-144)*^g^*	241 (118-578)*^j^*	**<**.**01**
	Visc	51 (7-86)	259 (175-390)*^k^*	**<**.**001**
T	Sc	627 (541-936)*^g^*	407 (316-629)*^l^*	.**02**
	visc	658 (623-1292)*^l^*	440 (164-853)*^h^*	**<**.**01**
A_4_	Sc	16 246 (12 369-22 667)*^g^*	12 561 (10 985-14 691)*^l^*	.15
	Visc	22 390 (20 414-29 517)*^l^*	18 529 (11 749-27 750)*^h^*	.16
DHEA	Sc	41 340 (35 970-66 337)*^g^*	35 010 (28 614-44 368)*^l^*	.20
	Visc	45 358 (36 878-79 301)*^l^*	40 113 (23 133-65 455)*^h^*	.29
Progesterone	Sc	1910 (1139-2883)*^g^*	1200 (1078-2190)*^l^*	.35
	visc	4482 (2167-8886)*^l^*	2878 (1855-4204)*^h^*	.15

Data are expressed as median (interquartile range). Boldface indicates statistical significance.

Abbreviations: A_4_, androstenedione; DHEA, dehydroepiandrosterone; E_1_, estrone; E_2_, estradiol; E_1_S, estrone sulfate; HT, hormone therapy; HT−, nonusers of HT; HT+, users of HT; T, testosterone.

^*a*^Independent-samples Mann-Whitney *U* test.

^*b*^n = 48.

^*c*^n = 47.

^*d*^n = 12.

^*e*^n = 26.

^*f*^n = 25.

^*g*^n = 11.

^*h*^n = 24.

^*i*^n = 42.

^*j*^n = 30.

^*k*^n = 44.

^*l*^n = 10.

Contrary to AT estrogen concentrations, the visceral AT concentration of T was approximately 30% lower in the HT+ compared to the HT− group (Table 3, Fig. 1). Although the concentration of total T in serum was comparable between nonusers and users of HT, serum concentration of free T was approximately 40% lower in HT users. The concentrations and AT to serum ratios of A_4_, DHEA, and progesterone were comparable between users and nonusers of HT. After comparing BMI-matched HT+ vs HT− subjects, only the small difference in subcutaneous AT T concentrations disappeared (627 vs 502 pmol/kg; *P* = .08; independent-samples Mann-Whitney *U* test; compared to Table 3).

The ratios of estrogens to androgens were 9- to 12-fold higher in subcutaneous, and 4- to 6-fold higher in visceral AT of HT users compared to nonusers (Table 4). In the HT+ group, subcutaneous AT also had a higher E_2_/E_1_ concentration ratio than visceral AT (0.06 vs 0.04, *P* = .02, n = 18).

**Table 4.** Ratios of estrogens and androgens in AT in users and nonusers of HT

	Subcutaneous adipose tissue		Visceral adipose tissue		*P*-valueHT−/+*^a^*
Ratio	HT−	HT+	HT−	HT+	Sc ATVisc AT
E_2_/E_1_	0.07 (0.01-0.09)*^b^*	0.06 (0.05-0.08)*^c^*	0.04 (0.02-0.07)*^d^*	0.04 (0.03-0.05)*^e^*	.57 .48
E_2_/T	0.11 (0.02-0.18)*^b^*	0.99 (0.30-1.85)*^f^*	0.08 (0.01-0.13)*^f^*	0.48 (0.30-1.45)*^g^*	**<.001<.001**
E_1_/A_4_	0.06 (0.06-0.08)*^b^*	0.67 (0.24-1.00)*^h^*	0.06 (0.04-0.08)*^f^*	0.26 (0.14-0.57)*^i^*	**<.001<.001**
E_1_ + E_2_/A_4_ + T	0.06 (0.06-0.11)*^b^*	0.70 (0.26-1.03)*^j^*	0.06 (0.04-0.08)*^f^*	0.27 (0.13-0.69)*^g^*	**<.001<.001**

Data are presented as median (interquartile range). Boldface indicates statistical significance.

Abbreviations: A_4_, androstenedione; E_1_, estrone; E_2_, estradiol; HT, hormone therapy; HT−, nonusers of HT; HT+, users of HT; Sc AT, subcutaneous adipose tissue; T, testosterone; Visc AT, visceral adipose tissue.

^*a*^Independent-samples Mann-Whitney *U* test.

^*b*^n = 11.

^*c*^n = 21.

^*d*^n = 13.

^*e*^n = 38.

^*f*^n = 10.

^*g*^n = 22.

^*h*^n = 9.

^*i*^n = 24.

^*j*^n = 8.

In general, serum and AT concentrations of E_1_ and E_2_ were higher, and the respective concentrations of T lower, with higher doses of E_2_ in the HT regimen (Fig. 1), especially for visceral AT E_1_ (*r* = 0.46, *P* < .01, n = 41) and E_2_ (*r* = 0.45, *P* < .01, n = 43). The ratios of E_2_/T, E_1_/A_4_, and E_1_ + E_2_/A_4_ + T (Table 4) in visceral AT also correlated positively with the dose of E_2_ in the HT regimen (*r* = 0.49, *P* = .02, n = 22; *r* = 0.56, *P* < .01, n = 24; and *r* = 0.57, *P* < .01, n = 22, respectively).

In the HT− group, serum concentrations of estrogens and E_1_ concentrations in subcutaneous and visceral AT correlated positively with measures of adiposity (Table 5). The estrogen/androgen balance, as represented by E_1_ + E_2_/T + A_4_, in subcutaneous and visceral AT correlated positively with BAI. In the HT+ group, the correlations of serum and visceral AT E_1_ with BMI and BAI, and of serum E_1_S with BMI, waist circumference, and BAI were inverse. The estrogen/androgen balance in subcutaneous and visceral AT showed a weak negative tendency in relation to BAI in the HT+ group (Table 5). Serum or AT concentrations of T, or AT concentrations of E_2_, did not correlate with any of the anthropometric measures in either users or nonusers of HT (data not shown).

**Table 5.** Correlations between serum and AT sex steroid concentrations and parameters of adiposity

	BMI	Waist circumference	BAI
HT−, n = 8-13			
Serum E_1_	0.52	0.48	**0**.**64***^a^*
Serum E_2_	**0**.**60***^a^*	0.51	**0**.**63***^a^*
Serum free E_2_	**0**.**64***^a^*	0.55	**0**.**65***^a^*
Serum E_1_S	0.33	0.41	**0**.**56***^a^*
E_1_ sc	**0.62** *^a^*	**0.65** *^a^*	**0.69** *^b^*
E_1_ visc	**0.80** *^c^*	**0.72** *^b^*	**0.65** *^a^*
E_1_ + E_2_/T + A_4_ sc	0.23	0.45	**0.62** *^a^*
E_1_ + E_2_/T + A_4_ visc	0.51	0.17	**0.70** *^a^*
HT±, n = 19-48			
Serum E_1_	**−0.35** *^b^*	−0.23	**−0.36** *^b^*
Serum E_2_	−0.22	−0.10	−0.29
Serum free E_2_	−0.14	−0.06	−0.22
Serum E_1_S	**−0.41** *^b^*	**−0.34** *^a^*	**−0.37** *^a^*
E_1_ sc	−0.17	0.08	−0.13
E_1_ visc	**−0.33** *^a^*	−0.24	**−0.39** *^a^*
E_1_ + E_2_/T + A_4_ sc	0.04	0.23	−0.10
E_1_ + E_2_/T + A_4_ visc	−0.13	−0.03	−0.21

Data are presented as Spearman correlation coefficient. Boldface indicates statistically significant correlation coefficients.

Abbreviations: A_4_, androstenedione; BAI, body adiposity index; BMI, body mass index; E_1_, estrone; E_2_, estradiol; E_1_S, estrone sulfate; Sc, subcutaneous adipose tissue; T, testosterone; Visc, visceral adipose tissue.

^*a*^ *P* < .05.

^*b*^ *P* ≤ .01.

^*c*^ *P* ≤ .001.

### Serum Leptin, Adiponectin, and Interleukin-6

Serum leptin (19 500 (9600-29 600) vs 14 500 (11 500-22 700) pg/mL (median (interquartile range); *P* = .43; independent-samples Mann-Whitney *U* test), adiponectin (11 [9-17] vs 12 [9-16] µg/mL; *P* = .98), and IL-6 (2.8 [2.4-4.3] vs 2.7 [1.8-8.5] pg/mL; *P* = .96) were similar between the HT− and the HT+ group. There were no correlations between the adipokine concentrations and subcutaneous or visceral AT concentrations of E_2_ (data not shown).

### STS Activity in AT

STS activity in converting E_1_S to E_1_ was slightly higher in subcutaneous AT of the HT− compared to the HT+ group (7.6 [7.0-8.3] vs 6.8 [6.5-7.1] nmol/kg AT/hour; mean [95% CI]; *P* < .01; Student *t* test). However, when comparing BMI-matched HT− and HT+ subjects, the significance was lost (7.9 [7.2-8.5] vs 6.8 [6.5-7.7] nmol/kg AT/hour; median [interquartile range]; *P* = .07; independent-samples Mann-Whitney *U* test).

Activity in visceral AT was similar between the 2 groups (7.5 [6.7-8.3] vs 7.0 [6.7-7.3] nmol/kg AT/hour; *P* = .08; independent-samples *t* test). STS activity did not correlate with estrogen concentrations in AT or with anthropometric parameters in either HT users or nonusers (data not shown).

### mRNA Expression of Genes for sex Steroid-metabolizing Enzymes in AT

**Figure 2.**
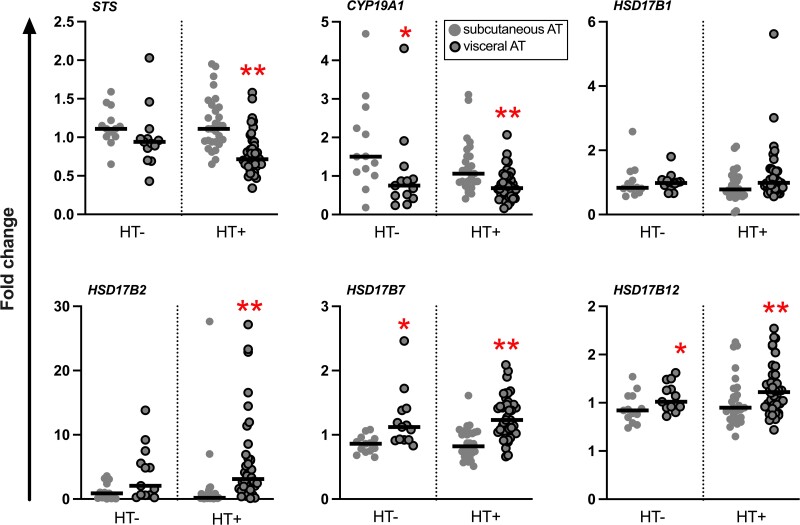
Relative mRNA expression levels of genes for sex steroid-metabolizing enzymes in abdominal subcutaneous and visceral AT. Results are presented as fold change. The black line represents the median. *P < .05; **P < .01, subcutaneous vs visceral AT (Wilcoxon signed-rank test, n = 12-13 for HT−, and 25-27 for HT+).

Abbreviations: *CYP19A1*, aromatase; *HSD17B1*, *HSD17B2*, *HSD17B7*, and *HSD17B12,* 17*β*-hydroxysteroid dehydrogenase 1, 2, 7, and 12; HT−, nonusers of HT; HT+, users of HT; *STS*, steroid sulfatase.

**Figure 3.**
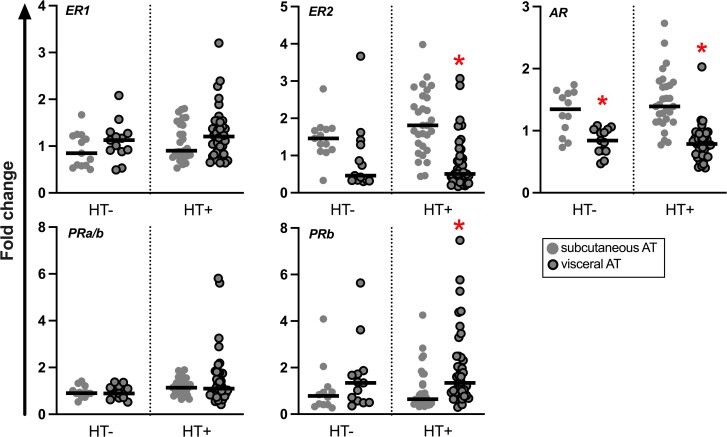
Relative mRNA expression levels of genes for sex steroid receptors in abdominal subcutaneous and visceral AT. Results are presented as fold change. The black line represents the median. *P < .05, subcutaneous vs visceral AT (Wilcoxon signed-rank test, n = 12-13 for HT−, and 25-27 for HT+).

Abbreviations: *AR*, gene encoding androgen receptor; *ER1,* gene encoding estrogen receptor α; *ER2*, gene encoding estrogen receptor *β*; HT−, nonusers of HT; HT+, users of HT; *PRa/b* and *PRb*, genes encoding progesterone receptors.

Overall, the mRNA expression pattern of genes for key enzymes involved in the metabolism of estrogens and androgens in AT, as well as for sex steroid receptor-encoding genes, were similar in the HT+ compared to the HT− group (Figs. 2 and 3). *STS* mRNA expression in visceral AT was higher in the HT− compared to the HT+ group, but when comparing BMI-matched HT− and HT+ subjects, the difference between the groups was lost (0.94 [0.78-1.05] vs 0.82 [0.62-1.07]; median [interquartile range]; *P* = .23; independent-samples Mann-Whitney *U* test).

In the HT− group, *STS* mRNA expression correlated positively with concentration of E_1_ and E_2_ in subcutaneous AT (*r* = 0.72, *P* < .01, n = 13; and *r* = 0.83, *P* < .01, n = 11, respectively). Also, *STS* mRNA expression level in visceral AT correlated positively with parameters of adiposity: BMI (*r* = 0.62, *P* < .05, n = 13) and waist circumference (*r* = 0.65, *P* < .01, n = 13). BAI correlated positively with the mRNA expression levels of *STS* in both subcutaneous and visceral AT (*r* = 0.63, *P* < .05, n = 13, and *r* = 0.77, *P* < .05, n = 13, respectively). The mRNA expression of *CYP19A1* in subcutaneous AT of the HT− group correlated positively with BMI (*r* = 0.67, *P* = .01, n = 13) and BAI (*r* = 0.71, *P* < .01, n = 13).

There were no significant correlations between gene mRNA expression levels studied and anthropometric parameters in the HT+ group.

## Discussion

The present study shows that postmenopausal women using per oral HT had markedly higher levels of E_1_ and E_2_ not only in serum but also in subcutaneous and visceral AT compared to women not using HT. The overall ratio of estrogens to androgens was higher in both subcutaneous and visceral AT in the HT+ compared to the HT− group. In visceral AT, the concentration of T in HT users was lower than in nonusers, and the dose of E_2_ in the HT regimen was correlated with higher ratios of estrogens to androgens in the visceral depot. Although this may at least to some extent mirror effects of exogenous per oral estrogen administration, the AT to serum ratios of estrogens were notably high also in the HT+ group, suggesting that these differences in AT sex steroid concentrations between users and nonusers of HT may also reflect altered local metabolism of sex steroids associated with HT use.

How oral menopausal HT affects the local metabolism of sex steroids in AT has, to the best of our knowledge, not been studied previously. Exogenous oral E_2_ is metabolized primarily in the liver, as well as in the bowel and in peripheral target tissues, mainly to E_1_, estriol, and their metabolites (41-43). E_1_S, the most abundant endogenous circulating estrogen in postmenopausal women, is an important metabolite of active estrogens formed for example in the liver (44). In the present study, users of HT had serum E_1_S concentrations that were 12 times higher than in nonusers of HT and also 3 times higher than those previously reported in premenopausal women (29), likely reflecting the effect of exogenous estrogen administration. The high concentration of E_1_S, along with its long half-life and slow clearance rate (45), renders it an important reservoir for the formation of estrogens in target tissues such as AT, where it can be taken up and hydrolyzed by the STS enzyme to active E_1_. In the present study, however, STS enzyme activity and mRNA expression levels of STS in subcutaneous and visceral AT were similar in users and nonusers of HT and would therefore be unlikely to provide the explanation for the high local AT E_1_ concentrations observed in HT users. In addition to E_1_S, STS hydrolyzes DHEAS to DHEA (36, 44), thus also affecting the local androgen balance available for aromatization.

The concept of sex steroid intracrinology builds on the premise that all active estrogens and most androgens in postmenopausal women are produced locally in peripheral target tissues using circulating precursor steroids as substrates, mainly DHEA and DHEAS of adrenal origin (46, 47). They are metabolically activated by a local AT steroid-metabolizing enzyme machinery, including aromatase and 17*β*-hydroxysteroid dehydrogenase enzymes (4, 46, 48). Also E_1_S can be taken up by AT and converted to E_1_ by STS and further to E_2_ by reductive 17*β*-HSD enzymes (6, 49). Whether AT production of estrogens and active androgens in peripheral tissues depends wholly on the supply of circulating C19 steroid precursors as substrates (50) or whether AT has the ability to synthesize sex steroids de novo from cholesterol (3, 51) remains a topic for further study. Intracrine production of estrogens and androgens, nevertheless, allows for high local sex steroid concentrations with local biological importance. In postmenopausal women, circulating concentrations of estrogens would then represent the residuals of locally produced estrogens from peripheral tissues, mainly AT, rather than being determinants of local tissue concentrations (33, 50, 52). This is supported by markedly high AT to serum ratios of E_1_ and E_2_ concentrations in postmenopausal women in the current study and in previous reports (5-7).

Aromatase is an important regulator of the local estrogen-androgen balance in AT, and aromatization of androgens to estrogens in peripheral tissues increases with age (53-56). Because of the limited amount of individual AT samples, we were not able to directly study the activity of aromatase or 17*β*-HSD enzymes. Ratios of estrogen to androgen concentrations can, however, be considered indirect estimates of aromatase activity in AT, and the E_2_/E_1_ ratio can be interpreted as an indirect estimate of reductive 17*β*-HSD enzyme activity (33). It has been shown that in severely obese premenopausal women, visceral adiposity and dysfunctional AT were related to an increased *CYP19A1* mRNA expression level and aromatase activity, assessed as the overall ratio of E_1_ + E_2_ to A_4_ + T, as well as higher inactivation of androgens, but not increased E_1_ and E_2_ concentrations (33). In the current study, we found the E_2_ + E_1_/T + A_4_ ratio to be higher in subcutaneous and visceral AT of HT users compared to nonusers, and in visceral AT, higher E_2_ + E_1_/T + A_4_ ratios were associated with higher doses of E_2_ in the HT regimen. *CYP19A1* gene mRNA expression levels in subcutaneous and visceral AT were similar in users and nonusers of HT. Although higher estrogen to androgen ratios observed in HT users do not directly prove induced aromatase activity in AT, it is possible that differences in aromatase activity could contribute to the observed differences in sex steroid concentrations in AT. This would need confirmation through directly measuring aromatase activity. The differences in local concentrations in comparison with nonusers of HT are, nevertheless, of such magnitude, with the AT to serum ratios for E_1_ and E_2_ remaining notably high, that it is unlikely they would merely reflect the effect of exogenous E_2_ administered in the HT regimen. This would support the previously published notion of intratissue estrogen changes being independent of circulating concentrations (50), even in the context of exogenous E_2_ administration.

The local balance between E_1_ and E_2_ reflects the activity of 17*β*-HSD enzymes, but as there are both reductive (E_1_ → E_2_) and oxidative (E_2_ → E_1_) forms, the balance is difficult to interpret. In the present study, there was no difference in the AT E_2_/E_1_ ratios between users and nonusers of HT. The concentrations of E_1_ in subcutaneous and visceral AT of women using HT were 4 to 7 times higher than in women not using HT, but also 2.5 to 5 times higher than the E_1_ concentrations previously described in AT of premenopausal women (29). The serum concentration of E_1_ in postmenopausal women with HT was also 1.5 times higher than in premenopausal women (29). For E_2_, the serum concentration in HT users amounted to 20% and the AT concentrations of E_2_ to 40% to 50% of the corresponding values in premenopausal women (29). Both in comparison with nonusers of HT as well as with premenopausal women, markedly high tissue levels of E_1_ in AT of HT users, especially in visceral AT, pose questions about their relevance in terms of the overall estrogen exposure in postmenopausal women, for example related to the risk for estrogen-dependent cancers in the breast or endometrium.

Androgens can exert their action directly or after conversion to estrogens. AT androgens in women can modulate local metabolism in terms of adipocyte differentiation and lipogenesis, adipokine signaling, and insulin sensitivity (57). T, the main active androgen, is produced in the ovaries or through conversion of A_4_ or DHEA in peripheral tissues. Overall, circulating androgen concentrations decline gradually with age, with no marked decrease at menopause (58). Sex steroids circulate mostly bound to SHBG and to albumin, with the albumin-bound and free fractions considered bioavailable (4). Hormonal contraception has been linked to a relative androgen insufficiency (59), and the same may be true for oral menopausal hormone therapy, through an increase in the hepatic production of SHBG and possibly a decreased production of adrenal and ovarian androgens (60). Although in our study, total serum T concentrations were similar in users and nonusers of HT, the lower serum concentration of free T in users of HT was likely a reflection of increased SHBG levels associated with exogenous E_2_. We also found that HT users had lower visceral AT T concentrations compared to nonusers of HT. It has previously been suggested that E_2_ could competitively inhibit the action of 3*β*-hydroxysteroid dehydrogenase in converting DHEA to A_4_ (61), but to what extent exogenous E_2_ actually lowers endogenous production of T and by which mechanisms is not well characterized (60).

The biological functions of progesterone are mainly associated with reproduction (62) and the role of the progestin component in EPT is to prevent detrimental growth of the endometrium associated with exogenous E_2_ administration (19). To our knowledge, this is the first study to assess the effect of different oral progestin components of EPT on sex steroid concentrations and metabolism in AT. Although the results of the present study suggest that exogenous administration of EPT has no evident direct influence on serum or AT concentrations of progesterone in postmenopausal women, it has previously been reported that synthetic progestins differ in their ability to bind to the progesterone receptor and other sex steroid receptors and may differ in their effects on for example cardiovascular health (63). We did not find differences in sex steroid concentrations when comparing users of ET and EPT, suggesting that the E_2_ component of HT is more relevant in terms of its effects on circulating and local AT sex steroid milieu.

We have previously shown that increased BMI in postmenopausal women not using HT was associated with increased local AT E_1_ concentration (6). The present study confirms this finding because AT concentrations of E_1_ increased with adiposity in the HT− group. In contrast, higher BMI and BAI were associated with lower serum and visceral AT E_1_ concentrations in users of HT. Contrary to Ofori et al (12), we did not observe a correlation between increases in anthropometric parameters describing body adiposity and serum T concentration in postmenopausal women with or without HT, nor with AT concentrations of T or E_2_ in either users or nonusers of HT. Data on the relationship between circulating T and adiposity in women have been conflicting, with some previous reports also reporting no clear correlation (64, 65).

We also assessed whether there would be changes in the mRNA expression levels of the genes encoding estrogen, androgen, and progesterone receptors associated with the use of HT, but overall, the mRNA expression profiles of genes for key sex steroid-metabolizing enzymes and sex steroid receptors were comparable between users and nonusers of HT. It is therefore likely that the differences evident in sex steroid concentrations described mostly result from posttranscriptional effects.

Our study has limitations. Because of the limited number of study subjects and scarcity of AT sample material, it was not possible to carry out all analyses for all subjects. For individual analyses, including for mRNA expression level data, the sample sizes were small. Negative results should therefore be considered with caution and would warrant larger sample numbers in future studies to confirm the results. The mRNA expression patterns for estrogen-metabolizing genes presented in the current study are, however, similar to the mRNA expression levels described earlier (6, 34, 36). AT samples were also insufficient for protein level measurement of sex steroid-metabolizing enzymes or for determination 17*β*-HSD or aromatase activities. Although we did not have the opportunity of using imaging techniques such as computed tomography for characterizing and quantifying AT depots, waist circumference and BAI are clinically useful predictors of visceral adiposity (17, 28). The subjects in the HT+ group had a slightly lower median BMI compared to subjects in the HT− group. This could be related to the treatment itself or be a difference between subjects before commencement of HT (66), but the effects of the BMI difference were mitigated by BMI-matching subjects from the 2 groups for between-group analyses. We observed high variability in the concentrations of E_2_ and E_1_ in the serum of women taking HT. Subjects included in the study did not report whether they had taken their daily dose of HT on the morning of recruitment to the study, which could be a factor explaining variable serum concentrations. Although higher doses of E_2_ in the HT regimen were associated with higher serum concentrations of E_2_ and E_1_, a larger study sample would be required to more carefully dissect other factors possibly underlying the variability in serum concentrations. This variability is, however, unlikely to have affected observed concentrations within AT. Studying metabolic markers such as blood glucose levels and circulating lipids in correlation with estrogen and androgen concentrations in both serum and AT could provide insight into the relationship between HT-induced changes in adipose tissue sex steroids and metabolic health. This remains a topic for further research.

Our study also has several strengths. The number of study subjects exceeded most previous studies studying AT sex steroid concentrations (5, 7, 33). Furthermore, to our knowledge, this is the first study to report local AT levels of estrogens, androgens, and progesterone in postmenopausal women with and without HT along with corresponding serum values. And finally, a major strength of our study is the use of specific and sensitive mass spectrometric methods for very accurate quantitation of a large range of sex steroids in both AT and serum.

To conclude, the current study describes for the first time the local AT milieu in terms of sex steroid concentrations associated with oral HT use in postmenopausal women, characterized by markedly high local concentrations and tissue to serum ratios of estrogens, and lower serum and visceral AT concentrations of T along with higher local estrogen to androgen ratios compared to nonusers of HT. In visceral AT, higher estrogen to androgen ratios were correlated with increasing dose of E_2_ in the HT regimen. In addition to direct effects of exogenous E_2_ administration, our results suggest intracrine enzymatic mechanisms in AT could be involved in the mitigating effect of HT on menopause-associated increase in android adipose tissue distribution.

## Data Availability

Some or all datasets generated during and/or analyzed during the current study are not publicly available but are available from the corresponding author on reasonable request.

## Abbreviations

A_4_: androstenedione
AT: adipose tissue
BAI: body adiposity index
BMI: body mass index
DEE: diethyl ether
DHEA: dehydroepiandrosterone
DHEAS: dehydroepiandrosterone sulfate
E_1_: estrone
E_1_S: estrone sulfate
E_2_: estradiol
EPT: estrogen-progestin therapy
ET: estrogen-only therapy
HT: hormonal therapy
LC-MS/MS: liquid chromatography-tandem mass spectrometry
LOQ = limit of quantification
STS: sulfatase
T: testosterone
